# Glioma Shapes Blood–Brain Barrier Integrity and Remodels the Tumor Microenvironment: Links with Clinical Features and Prognosis

**DOI:** 10.3390/jcm11195863

**Published:** 2022-10-04

**Authors:** Xiaokai Li, Lei Li, Ke Zhou, Huixiang Zhang, Ali Abdi Maalim, Xingyu Chen, Ximiao He, Xinmin Ding, Chuanrui Xu, Yonghong Wang

**Affiliations:** 1 Department of Oncology, Affiliated Hospital of Guizhou Medical University, Guizhou Cancer Hospital, Guiyang 550008, China; 2 School of Pharmacy, Tongji Medical College, Huazhong University of Science and Technology, Wuhan 430030, China; 3 Hanyang 3rd School, Wuhan Economic & Technological Development Zone, Wuhan 430030, China; 4 Department of Medical Oncology, The First Affiliated Hospital of Zhengzhou University, Zhengzhou 450052, China; 5 Department of Neurosurgery, Tongji Hospital, Tongji Medical College, Huazhong University of Science and Technology, Wuhan 430030, China; 6 Department of Physiology, School of Basic Medicine and the Collaborative Innovation Center for Brain Science, Tongji Medical College, Huazhong University of Science and Technology, Wuhan 430030, China; 7 Department of Neurosurgery, Shanxi Bethune Hospital, Shanxi Academy of Medical Sciences, Tongji Shanxi Hospital, Third Hospital of Shanxi Medical University, Taiyuan 030032, China

**Keywords:** glioma, blood–brain barrier, tumor microenvironment, prognosis

## Abstract

Background: The presence of the blood–brain barrier (BBB) uniquely distinguishes the brain from other organs, and various brain pathologies, including cancer, can disrupt or breach the BBB. The specific implications of BBB alterations in glioma have not been sufficiently clarified. Methods: In this study, statistical analysis of the TCGA pan-glioma dataset and four other validation cohorts was used to investigate the infiltration of BBB constituent cells (endothelial cells, pericytes and astrocytes) in the glioma tumor microenvironment (TME). Results: We found that the infiltration proportions of the three BBB constituent cell types were highly collinear, which implied alteration of the BBB. Hence, we developed an index, the BBB score, which is calculated based on the infiltration proportion of BBB constituent cells. Furthermore, we observed that patients with higher BBB scores were more likely to be diagnosed with more malignant entities in the TCGA database according to significant molecular features, such as IDH mutation status and 1p/19q deletion. The BBB score was also strikingly positively correlated with WHO grade in other cohorts. More importantly, a higher BBB score correlated with shorter survival time and unfavorable prognosis in glioma patients. Finally, we showed that TME-related pathways may regulate BBB alterations and that coinhibitory immune checkpoints were enriched in samples with higher BBB scores. Conclusions: We showed that TME-related pathways may regulate BBB alterations and that coinhibitory immune checkpoints were enriched in samples with higher BBB scores. Assessing BBB alterations may help elucidate the complex role of the glioma TME and suggest new combination treatment strategies.

## 1. Background

Glioma remains the most widespread primary brain malignancy, and its incidence in children aged 0 to 14 years has risen significantly, according to recent statistical reports [1,2]. In addition, more than half of gliomas in adults are classified as glioblastoma multiforme, which is the deadliest form of brain tumor, with no approved targeted drugs and a median survival of 12.6 months [3]. In addition to efforts to gain deeper insights into the genetic and phenotypic variability within glioma, researchers in the field have also paid increasing attention to the distinctive ecosystem in which cancer cells can survive and proliferate [4,5].

The tumor microenvironment (TME) contains various types of noncancerous cells in addition to tumor cells, including fibroblasts, endothelial cells and immune cells [6,7]. Although some of these cell types are prevalent in brain malignancies, they also display a series of unique properties that distinguish the normal brain from other physiological tissues and organs [8]. The blood vessels in the central nervous system (CNS), termed the blood–brain barrier (BBB), play a pivotal role in these distinct properties. CNS endothelial cells (ECs) are the central component of the BBB, and they tightly regulate the chemical microenvironment of the CNS, which is important for maintaining brain homeostasis and for neural protection. Apart from endothelial cells, neural stem cells, pericytes and astrocytes also participate in brain homeostasis and together block the transport of approximately 98% of molecules between the systemic circulation and the brain.

The brain is physically protected from inflammation by the BBB, so it was long regarded as an “immune privileged” organ [9,10]. However, accumulating evidence indicates that BBB dysfunction is associated with a massive infiltration of diverse immune cells [11], which may be explained by the loss of BBB integrity leading to leakiness and a dysfunctional blockade [12]. While the BBB confines the access of many molecules and a variety of cells, it is not enough to serve as an impenetrable barrier to the invasion and migration of cancer cells [13]. Therefore, metastatic cancer cells leverage multiple approaches to cross the physiological layers, resulting in functional changes in BBB constituent cells and consequently heterogeneous BBB integrity within brain tumors [14,15]. Accordingly, there could be distinct interactions and bidirectional regulation between the BBB and the glioma TME. An integrated analysis will improve understanding of the multidimensional regulatory mechanisms underlying the development of glioma and open up new perspectives regarding their significance for basic research and clinical application.

Similar to the case of BBB constituent cells, little is known about the roles of neural lineage cells (neurons and oligodendrocytes) in glioma. Moreover, the vast majority of nontumor cells in the TME, microglia, which are derived from immature yolk cells, are the main innate immune cells in the brain. However, microglia can create a supportive stroma for tumor cell expansion and invasion [16,17]. All nontumor cells can be manipulated by cancer cells in the glioma TME [18], and the infiltrating proportion of BBB constituent cells in the TME can be deemed to be the cause of the imbalance in and deficient integrity of the BBB.

Here, the GSVA (Gene Set Variation Analysis) algorithm [19] was applied to digitally dissect the glioma TME in our study, and six stromal cell types (endothelial cells, astrocytes, pericytes, microglia, neurons and oligodendrocytes), all of which are residents in the brain and hijacked during glioma tumorigenesis [18], were chosen for analysis. Statistical analysis of TCGA glioma patients and additional cohorts revealed the linearity of the dynamics of three cell types that compose the BBB (endothelial cells, astrocytes, pericytes) in the tumor TME. Then, the defined BBB score and GSVA scores of each stromal cell type were found to be related to the progression of glioma. According to the association between those scores and significant clinical features, we further highlight the outstanding power of the BBB score to predict the survival of glioma patients in all cohorts. Finally, bioinformatics analysis indicated that extracellular matrix organization and related pathways might mediate the alteration of the BBB, along with the immunosuppression and immune evasion phenotypes. In summary, our integrated and comprehensive study introduce the novel view that BBB alterations in the context of the dynamic glioma microenvironment should be fully evaluated for more robust classification and clinical prediction of glioma.

## 2. Materials and Methods

### 2.1. Public Data Collection

For the TCGA glioma cohort, we chose low-grade glioma (TCGA-LGG) and glioblastoma (TCGA-GBM) patients; normalized level three RNA sequencing data and survival information was downloaded from The UCSC Cancer Genomics Browser (https://tcga.xenahubs.net/; accessed on 15 October 2018). Other detailed clinical and molecular information for both LGG and GBM patients was obtained from published literature [20]. Other independent cohorts were obtained from the GEO database accessed on 6 May 2019 (https://www.ncbi.nlm.nih.gov/gds). For validation, four groups of glioma patients (GSE16011 [21], REMBRANDT [22], GSE4290 [23] and GSE4412 [24]) were enrolled in our study, and we directly downloaded the corresponding series matrix files from the web page. When multiple probes matched a gene in the array data, the probe with the median value was selected. All available clinical data were extracted from the original downloaded files. Detailed information for all cohorts and the important clinical features of their patients are shown in Table S1. Finally, the mRNA expression matrix of all samples in the TCGA project and the associated cancer type information were also downloaded from https://tcga.xenahubs.net/; accessed 15 October 2018.

### 2.2. Calculating the Infiltration of Stromal Cells and BBB Score

To infer the stromal cell infiltration degree in the glioma TME, we used the GSVA algorithm [19]. The gene signature from each type of stromal cell (endothelial cells, astrocytes, pericytes, neurons, fibroblasts, epithelial cells, hepatocytes and melanocytes) came from the publication xCell [25], and the first six cell types were systematically analyzed in our study. As more than one signature existed for a cell type, we retained only the signature that included the most genes. Signatures for the cell types (microglia and oligodendrocytes) not included in xCell could be found in the web resource CellMarker accessed on 23 May 2019 [26] (http://biocc.hrbmu.edu.cn/CellMarker/). The infiltration degree of each type of stromal cell was defined using a standardized score ranging from −1 to 1, and this numeric value implied the proportion of the cell type in the TME; that is, a higher GSVA score indicated a greater prevalence of that cell type in the TME. We defined the average values of the GSVA scores of three kinds of stromal cells (endothelial cells, astrocytes, and pericytes) as the “BBB score” in our study; this score could represent the alteration of the blood–brain barrier in brain pathologies (the involved genes are listed in Table S2).

### 2.3. Bioinformatic Analysis

The stromal scores and tumor purity of the glioma samples were computed with an R package [27], and Pearson correlation and principal component analysis were used to illustrate their relevance to the GSVA score of different stromal cells. Genes whose FPKM values were most positively correlated with BBB score (rho > 0.7) were submitted for Gene Ontology (GO) biological process pathway analysis by the clusterProfiler R package [28]. Extracellular matrix (ECM) genes were selected as described in a previous publication [29], and the ECM genes whose expression levels were most positively and negatively correlated with BBB score in the TCGA cohort were determined. Immune checkpoint molecules were obtained from another publication [30], and we normalized each quotient (log10 + x) of fifteen pairs of immunity genes instead of the binary indicators and summed them as a score (IMPRES) in glioma patients. The infiltration of different kinds of immune cells in the TME was inferred with a reported method [31], and the gene set used in our study can be found in Table S3.

### 2.4. Statistical Analysis

Associations between BBB score and continuous variables (age and KPS) were tested by using Spearman correlation analysis. Differences in BBB score or other stromal cell GSVA scores between groups according to other clinical features were evaluated by T test or one-way ANOVA. We performed survival analysis with respect to the BBB score and six kinds of stromal cell GSVA scores by using the Kaplan–Meier estimator and Cox proportional hazard model, and the log-rank test was conducted to evaluate the statistical significance between different groups. Time-dependent ROC analysis was performed with the survival ROC R package [32] in both the testing and validation cohorts. The classical ROC curve was applied as the quality metric of their ability to predict the subtype of glioma. Patients with missing clinical information were excluded from the study. All statistical analyses were conducted using R 3.5.1 and SPSS version 16.0, and a *p* value less than 0.05 was regarded as statistically significant.

## 3. Results

### 3.1. Different Relationships between the Infiltration of Stromal Cell Types with Fractions of Stroma and Glioma Purity

To confirm the accuracy of cellular composition determination from bulk tumor samples with the GSVA algorithm, we leveraged the broad mRNA expression data of TCGA pan-cancer samples, including more than 10,000 patients and 33 cancer types. We found that much higher scores of brain-specific cells (neurons and oligodendrocytes) were observed in nervous system tumors (LGG, GBM and PCPG) (Supplementary Figure S1A,B). However, fibroblasts and epithelial cells, which are generally present in most solid tumors, were estimated to have extremely low scores in glioma, as well as in blood cancers (LAML and DLBC) (Supplementary Figure S1C,D). As further convincing evidence, hepatocytes and melanocytes exhibited the highest scores in HCC and SKCM, respectively, in our analysis (Supplementary Figure S1E,F). Together, these results verified the validity and precision of the approach taken in our study to infer the proportion of nontumor cells infiltrating the TME.

**Figure 1:**
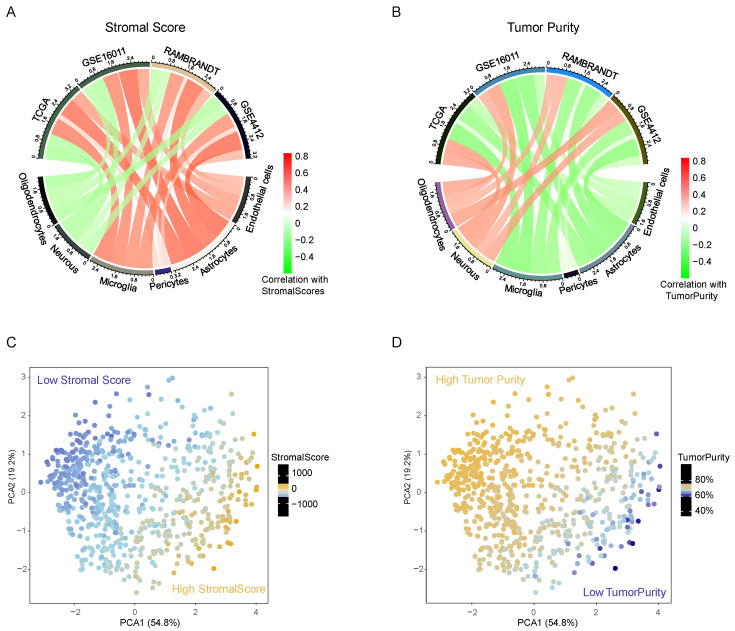
The remarkable but distinct correlations between the infiltration of six kinds of stromal cells with stromal scores (**A**) or tumor purity (**B**). Red represents a positive correlation, and green represents a negative correlation among all cohorts. PCA revealed that the GSVA scores of stromal cells could distinguish the numerical value of stromal scores (**C**) or tumor purity (**D**).

Distinct stromal cells build a complex and unique ecosystem of brain malignancy. Therefore, we evaluated a catalog of six cells (endothelial cells, astrocytes, pericytes, microglia, neurons and oligodendrocytes) based on the sequencing data of bulk tumor tissue in TCGA panglioma, consisting of 529 low-grade glioma patients and 156 glioblastoma patients. Recently, Yoshihara and other researchers have developed a universal method, “ESTIMATE”, to quantify the infiltration of stromal and immune cells and estimate tumor purity, according to transcriptomic data from tumor samples [27]. In our study, we found a distinct relationship between the GSVA score of each stromal cell type and the stromal scores calculated by the ESTIMATE R package. In the TCGA database, the infiltration of endothelial cells, astrocytes, pericytes and microglia had an obviously positive correlation with the total fraction of stromata, but neurons and oligodendrocytes exhibited completely opposite outcomes, and all results were markedly validated in other databases (Figure 1A). In addition, these observations were thoroughly reversed when comparing the stromal cell content and tumor purity (Figure 1B). Then, we conducted principal component analysis (PCA) of the six stromal cell GSVA scores and the results showed that both the total fraction of stromata and tumor purity could be markedly segmented by the first two components. Together, these results hinted at the unique features of neurons and oligodendrocytes, which more frequently existed in purer glioma samples. All stromal cells synergetically constituted the complex glioma TME and reflected the infiltrative pattern of noncancerous cells in the TME and the varying purity levels of the glioma tissue.

### 3.2. Association between BBB Alterations and Clinical and Molecular Characteristics in the TCGA Cohort

**Figure 2:**
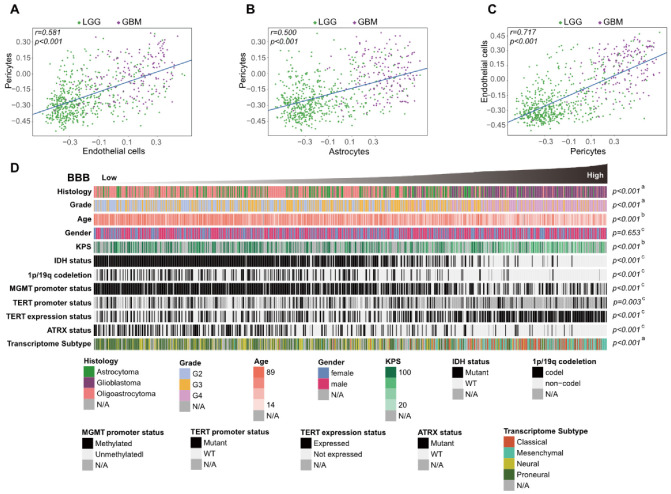
Correlation of GSVA scores between BBB constituent cells (**A**–**C**). Glioma patients in the TCGA cohort were arranged in order of increasing BBB score (**D**), and the relationships between different patient characteristics and BBB score was evaluated (a, The distribution of BBB scores among several groups was assessed using one-way ANOVA. b, The associations between BBB score and continuous variables was assessed using Spearman correlation tests. c, The distribution of BBB scores between two groups was assessed using Student’s *t* test).

To explore the intrinsic relevance and stability in the glioma TME of the six cell types in the catalog, we generated a correlation coefficient matrix of their GSVA scores. In keeping with the correlation between the content of a single cell type and the total stromata in the TME, two subgroups of these six cells could be identified (Supplementary Figure S2A–E). Interestingly, a considerably positive correlation between the GSVA scores of BBB constituent cells (endothelial cells, astrocytes and pericytes) was detected in TCGA patients (Figure 2A–C), and this discovery was also confirmed in GEO database patients (Supplementary Figure S2B–E). Considering that the change in the infiltration proportion of these cells could reflect the degree of aberrance of BBB integrity and function, we developed a BBB score derived from the average GSVA scores of these cell types. Then, glioma patients from the TCGA cohort were arranged in order of increasing BBB score, and we evaluated the distribution of subgroups according to their significant clinical features (Figure 2D). We further found that there were higher BBB scores in glioblastoma than in other histology types or patients with lower grades, and positive correlations between BBB score and age at diagnosis or Karnofsky performance score (KPS) were also noted. In particular, IDH mutation and 1p/19q codeletion conferred lower BBB scores, but TERT promoter mutation was not associated with notable BBB score elevation. We also explored the relationships between BBB score and other factors, such as other molecular classifications, and all the results indicated that more aggressive gliomas were characterized by higher BBB scores in the TCGA cohort.

### 3.3. High BBB Score Indicates Glioma Progression in the Validation Cohorts

**Figure 3:**
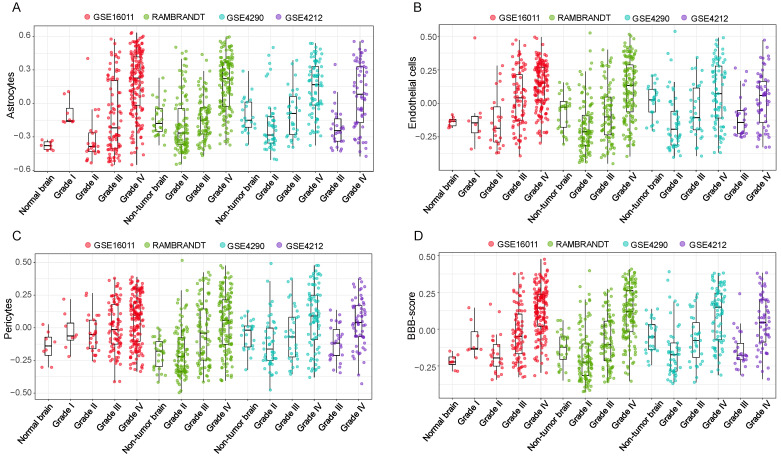
Distinct distribution of astrocytes (**A**), endothelial cells (**B**), pericytes (**C**) and BBB scores (**D**) according to glioma WHO grade in four independent cohorts.

Based on the results of the TCGA cohort, which showed that patients with higher BBB scores were more likely to have more malignant characteristics, such as wild-type IDH status, 1p/19q noncodeletion and TERT mRNA expression [33], we could conclude that the BBB score would increase with the malignant progression of glioma. As WHO grade is commonly regarded as one of the crucial clinical features of glioma patients, we analyzed the associations between BBB score and WHO grade in four GEO cohorts (GSE16011, REMBRANDT, GSE4290 and GSE4412) to test our assumption. Consistent results were observed in all independent validation cohorts: the GSVA scores of three BBB constituent cells and the BBB score presented increasing trends from low-grade to high-grade glioma patients (Figure 3A–D). We also conducted parallel analysis of other stromal cells and profiled the distinct patterns of neuron and oligodendrocyte GSVA scores with increasing grade (Supplementary Figure S3A–C). Together, these results revealed the possibility that BBB alterations participate in the progression and deterioration of glioma and highlighted the potential value of BBB score for more precise clinical classification.

### 3.4. The Outstanding Performance of BBB Score for Survival Prediction

**Figure 4:**
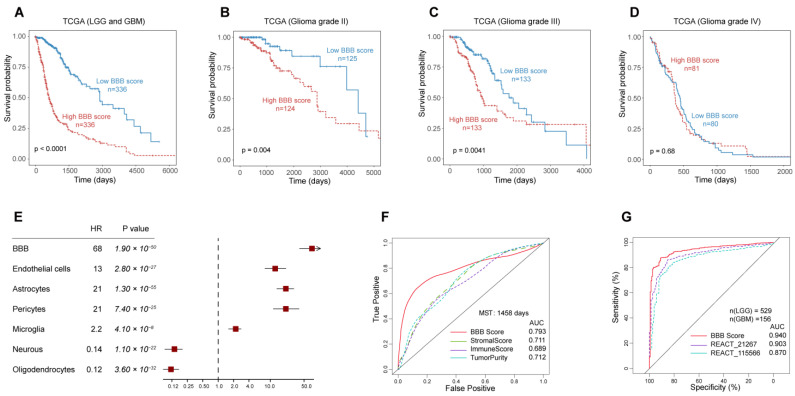
The prognostic value of the BBB score in the TCGA cohort (**A**) and subgroups defined by WHO grade (**B**–**D**). The association between GSVA scores of all stromal cells in the glioma TME with clinical outcome (**E**) and BBB score had the most robust predictive and discriminative power for median survival time (**F**) and WHO grade (**G**) of TCGA glioma patients.

Dichotomization dependent on the median BBB score value and Kaplan–Meier curves were harnessed for survival analysis of glioma patients, and parallel analysis was implemented in all TCGA patients or according to different WHO grades. We found that patients with lower BBB scores had markedly longer overall survival times than those with higher BBB scores among grade II, grade III and all patients in the TCGA cohort, although this pattern was not observed specifically among patients diagnosed with grade IV disease (Figure 4A–D). Furthermore, we used the Cox proportional hazard model to estimate the prognostic impact of all stromal cells in the total TCGA patient cohort. The forest map showed that only more neuron and oligodendrocyte contents in the glioma TME could predict a favorable prognosis (Figure 4E). The prognostic impact of six stromal cell GSVA scores and BBB score was validated in the same way (Supplementary Figure S4A–C). Less robust relationships between high GSVA scores of the three BBB constituent cells or BBB score and risk were observed in the GSE4412 cohort, which could be accounted for by higher grade cases (grade III and grade IV) and the extremely poor prognosis of patients in this cohort. Univariate and multivariate Cox regression also confirmed that BBB score, in addition to other known clinical features, was an independent prognostic indicator in glioma (Table S4).

Next, we investigated the specificity and sensitivity of BBB score in the prediction of survival with the median survival time of the TCGA cohort and compared BBB score with previously proposed indicators [27] (stromal scores, immunity scores and glioma purity) by analyzing the ROC curves. BBB score displayed superior predictive validity, with an AUC of 0.793, higher than those of other factors (Figure 4F), and in three other independent cohorts, BBB score maintained its outstanding performance for survival prediction (Supplementary Figure S5A–C). Subsequently, we examined the prediction of low- or high-grade subtypes in glioma as a quality metric, and the results indicated strikingly that BBB score performed better than other reported pathway GSVA scores in the TCGA cohort [20] (Figure 4G). In addition to patient survival status, we reviewed the association between BBB score and the recurrence of glioma and found that recurrent and secondary low-grade glioma patients showed higher BBB scores than glioblastoma patients (Supplementary Figure S6A,B). Moreover, there was also a moderate negative correlation between BBB score and days to recurrence (Supplementary Figure S6C).

### 3.5. Extracellular Matrix Genes Link High BBB Score to Immunosuppression and Immune Evasion

**Figure 5:**
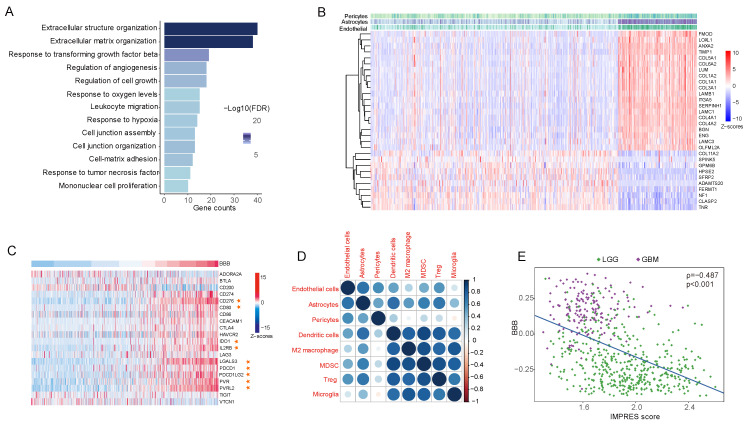
Tumors with higher BBB scores exhibit reorganization of the extracellular matrix and deficiency of the tumor immune response. (**A**), ECM (extracellular matrix)-relevant processes were the main biological function implicated among genes that were positively correlated with BBB score. (**B**), The top 20 and top 10 genes most positively and negatively correlated with BBB score. (**C**), The relationships between BBB score and coinhibitory immune checkpoint genes. (**D**), Dot plot showing the correlation coefficient matrix of BBB constituent cells and selected immune cell GSVA scores. (**E**), Scatter plot indicating that higher BBB scores indicate lower IMPRES values in TCGA glioma patients.

To explore the biological significance and molecular signaling pathways associated with the infiltration of BBB constituent cells in the TME, Spearman correlation analysis was carried out to identify genes linked to BBB score from the mRNA expression matrix in the TCGA database. Genes most positively correlated with BBB score (rho > 0.7) were selected for GO (Gene Ontology) analysis. The enriched biological process pathways showed ECM remodeling and TGF-β signaling-mediated alteration of the BBB (Figure 5A), and these conclusions were broadly reinforced by similar analyses in other GEO datasets (Supplementary Figure S7A–D). Moreover, we identified upregulated and downregulated ECM genes corresponding with the infiltration of BBB constituent cells (Figure 5B) from among 249 reported cancer-associated ECM genes [29].

Tumor matrix dynamics and specific stromal cells can shape the complicated tumor ecosystem and may function in regulating each step of the cancer–immunity cycle and participate in immune evasion [34,35]. In addition to ECM genes, a positive correlation between BBB score and immunity checkpoints (PD-1, PD-L1, CTLA-4 and B7-H3) in the TCGA database was also observed (Figure 5C), and this positive correlation persisted when comparing BBB constituent cells with cell types (dendritic cells, M2 macrophages and so on) that promoted the dysfunctional differentiation of cytotoxic T lymphocytes [18]. Finally, patients with higher BBB scores had evidently lower scores on IMPRES, a predictor of response to immune checkpoint blockade-based tumor therapies [30]. In conclusion, the results suggested that the infiltration of endothelial cells, astrocytes and pericytes and BBB alterations in the TME were linked to immunosuppression and immune evasion phenotypes in glioma.

## 4. Discussion

There is a deepening understanding that gliomas are complex and heterogeneous malignancies composed of neoplastic and non-neoplastic cells, the latter of which can individually or synergistically contribute to tumor formation, progression and discrepant responses to treatment. It is also becoming clearer that non-neoplastic stromal cells in the TME vary from primary and metastatic brain malignancies to other solid tumors due to the unique properties and distinct features of this organ [36,37]. However, the extent of their specificity and heterogeneity, how they shape the glioma TME, and the explicit approaches by which they communicate with cancer cells remain poorly characterized. In our study, we portrayed the landscape of glioma non-neoplastic cells in the TME based on the infiltration of six dominant stromal cell types [8,18] and identified different patterns according to the association between the content of each cell type in the TME and the total fraction of stromata, glioma purity, WHO grade and prognostic condition of glioma patients. These results highlighted the meaningful and distinct roles of each stromal cell type in glioma biology, partly reflecting the complex glioma microenvironment, and offering new perspectives into the clinical management of gliomas.

The blood–brain barrier (BBB), a unique physiological structure that commonly exists in mammals, protects the normal brain by blocking the vast majority of materials and molecules from accessing brain tissue. The BBB determines the distinctive composition of the ECM in the brain as well as important features of the glioma TME. Abnormal disruption of the BBB could result in neuronal degenerative changes or other non-neurological diseases, and loss of BBB integrity leads to leakiness and blockade failure, which further remodel the brain TME and provide a microenvironment that facilitates cancer cell survival; this is frequently observed in glioma patients [12]. The key insight of our study was the close association between the high infiltration of BBB constituent cells and a malignant phenotype in glioma regardless of genomic, clinical, and biological conditions, implying that BBB alteration would more directly accelerate the progression of glioma through various modes. In addition, the outstanding performance of the BBB score for survival prediction and the link between high BBB score and deficiency of the tumor immune response suggested that this indicator developed in our study should be fully utilized for accurate classification and clinical prediction.

Noncancerous cells existing in the TME include stromal cells and multiple immune cells, which together dilute the purity of tumor tissues and play important roles in tumor biology, so tumor purity is regarded as a microenvironmental factor and has received substantial attention from oncologists [38,39]. In recent years, several computational methods have been developed to infer tumor purity based on bulk or targeted genomic sequencing data of tumor samples [40,41,42]. The ESTIMATE algorithm [27] was utilized in our study owing to the availability of RNA-seq and microarray data from public databases. We systematically analyzed the associations between the infiltration proportion of each stromal cell type with the total fraction of stromata in the TME and glioma purity and found that there was not a universal positive or negative correlation among any cell types. A higher purity and lower stromata fraction of glioma seemed to confer more oligodendrocyte content in the TME, and higher oligodendrocyte GSVA scores belonged to patients with regressing gliomas consistent with a lower grade, favorable prognosis and longer survival time. Other researchers have reported that endogenous oligodendrocytes are capable of repressing the growth and proliferation of glioblastoma cells by paracrine signaling via WNT inhibitory factor 1, and this explanation strongly supports the seemingly paradoxical result in our study.

In addition, the infiltration of neurons in the TME had a pattern similar to that of oligodendrocytes, which is likely the cause of some misleading reports that neuronal activity could affect glioma growth and invasion [43,44,45]. Nevertheless, we noticed that the interaction and communication of cancer cells with neurons did not occur through direct contact but rather was mediated mainly by excitatory synapses [18], so infiltration of neurons in the TME is not necessarily equivalent to neuronal activity, and the GSVA score of neurons only indicated the existence of neurons in the TME rather than the secretion of neurotransmitters and synaptic signaling. Furthermore, excessive glutamate release by glioma cells has profound neurotoxicity [46], and the TME of aggressive glioma is a severe environment with high vascularization and striking hypoxia; it could be supposed that neural lineage cells could not survive for the long term in such conditions.

Unlike the case for neurons and oligodendrocytes, there was a consistent positive correlation between other cell GSVA scores and stromal scores. Among the four stromal cell types, microglia are known as a unique type of tumor-associated macrophage and often comprise up to approximately thirty percent of the glioma mass [47]. These cells were deemed to have an intimate relationship with tumor cells and to facilitate glioma proliferation, survival and migration in various manners [5]. In our study, we also confirmed that a higher microglial content in the TME was accompanied by a higher WHO grade and predicted poor prognosis in almost all cohorts.

For the remaining stromal cells (endothelial cells, astrocytes and pericytes), less is known about the function of these cells in the TME, relative to microglia, and their crosstalk with tumor cells reminds us of the BBB alterations in the glioma TME. We uncovered a positive correlation between their GSVA scores and the BBB score developed in our study, similar to the case for stromal scores and tumor purity. This score was associated with more malignant molecular and clinical features of glioma patients. More importantly, BBB score had better performance in survival prediction and could also distinguish the status of glioma recurrence after surgical resection. Based on bioinformatics analysis, we found that activation of TGF-β signaling and ECM gene dysregulation regulated the infiltration of BBB constituent cells, similar to the existence and function of fibroblasts in other solid tumor microenvironments [29].

Advances in single-cell RNA sequencing (scRNA-seq) have allowed us to investigate the nature and extent of intratissue diversification [35,48,49], particularly for highly heterogeneous and complex tumor ecosystems. Unfortunately, there has been no use of scRNA-seq to analyze the stromal and immune cell profiles in the glioma TME to date; our inferences based on bulk tumor RNA sequencing data should be validated by more precise scRNA-seq analyses in the future. Although our study presented a relatively rough primary stromal cell catalog, it still systemically clarified a missing link between the BBB, the TME, and other microenvironmental factors. Therefore, BBB alterations as well as glioma-relevant nontumor cells within the microenvironment confer important clinical and biological effects. This knowledge could be used to guide clinical prediction and classification and provide more treatment options for glioma patients.

## 5. Conclusions

We showed that TME-related pathways may regulate BBB alterations and that coinhibitory immune checkpoints were mainly enriched in samples with higher BBB scores. Further assessing BBB alterations may help elucidate the complex role of the glioma TME and suggest new combination treatment strategies.

## Data Availability

Validation data that support the findings of this study have been deposited in the GEO database accessed on 6 May 2019 (https://www.ncbi.nlm.nih.gov/gds) with the accession codes “GSE16011”, “RAMBRANDT”, “GSE4290” and “GSE4412”.

## Supplementary Materials

The following supporting information can be downloaded at: https://www.mdpi.com/article/10.3390/jcm11195863/s1.

## Abbreviations

BBB: blood–brain barrier; TME: tumor microenvironment; CNS: central nervous system; GSVA: gene set variation analysis; ECM: extracellular matrix; KPS: Karnofsky performance score.
