# Supplementary material for: Glioma Shapes Blood–Brain Barrier Integrity and Remodels the Tumor Microenvironment: Links with Clinical Features and Prognosis
Source: J Clin Med. 2022 Oct 4;11(19):5863. doi: 10.3390/jcm11195863 (PMC9570525; doi:10.3390/jcm11195863)
Supplement: Supplementary file 1 [file jcm-11-05863-s001.zip › jcm-1852459-Supplementary Figures-1.pdf]

Figure S1

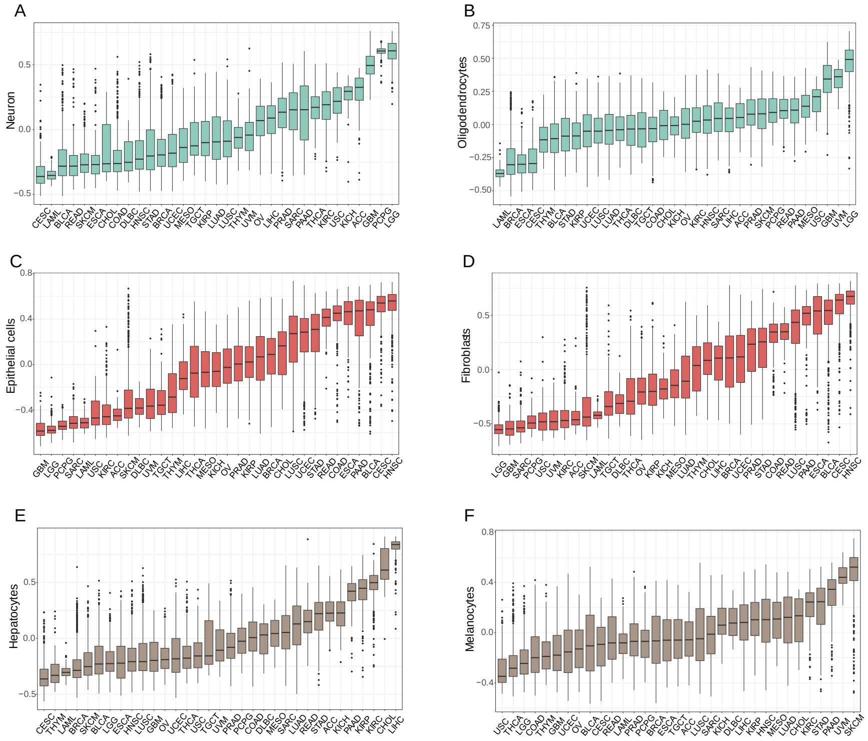

Figure S1: The infiltration of stromal cells in different tumor types. In TCGA pan-cancer, GSVA scores of neurons(A), oligodendrocytes(B), epithelial cells(C), fibroblasts(D), hepatocytes(E) and melanocytes(F) are distributed differently. Different tumor types are arranged in order from small to large by the median of GSVA scores of each stromal cell.

Figure S2

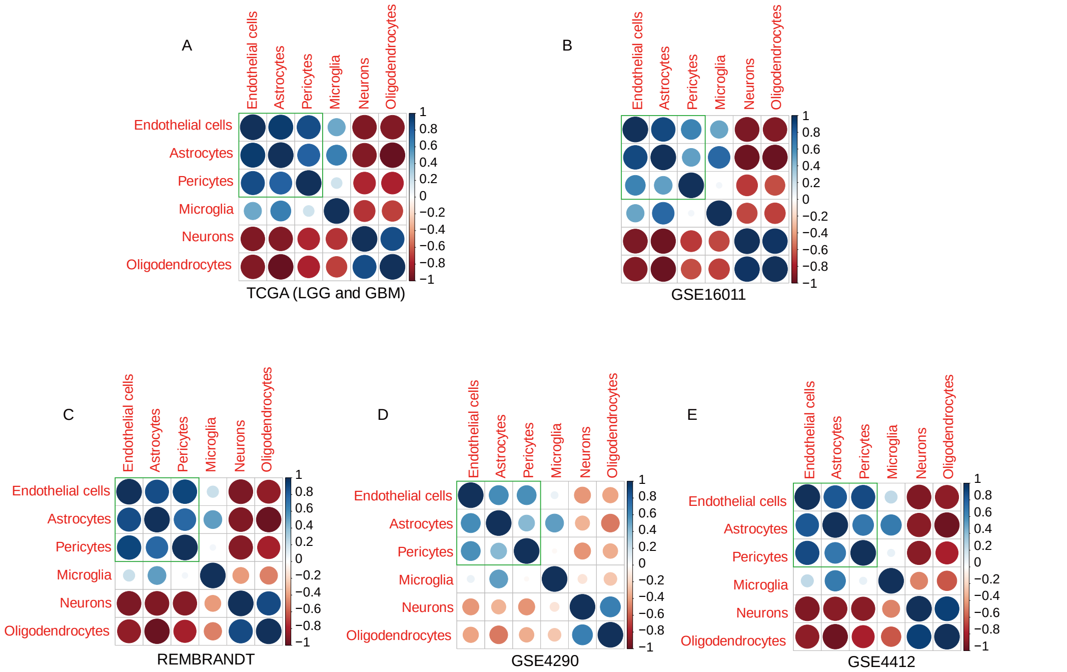

Figure S2: Correlation coefficient matrix of GSVA score of six kinds of stromal cells. They were analyzed in five independent glioma

cohorts, TCGA(A), GSE16011(B), REMBRANDT(C), GSE4290(D) and GSE4412(E). The blue represents positive correlation, the red represents negative correlation, and the green box represents the collinearity of GSVA scores of endothelial cells, pericytes, and astrocytes.

Figure S3

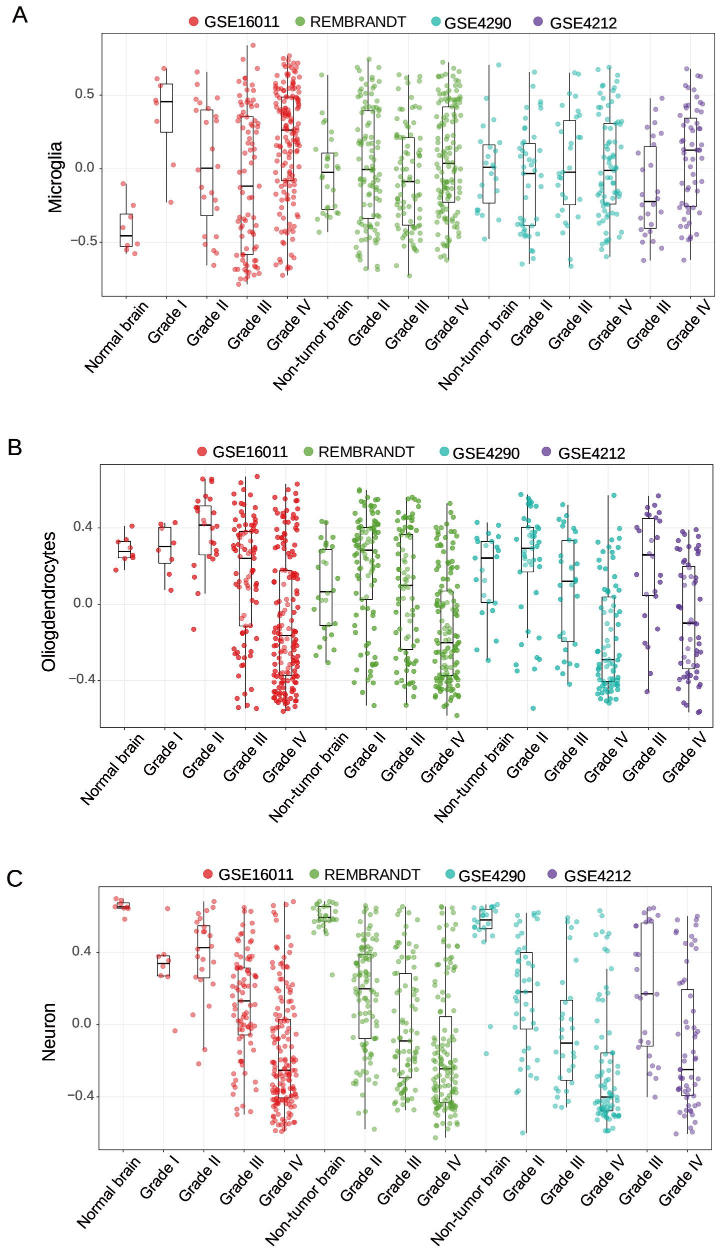

Figure S3: GSVA scores of microglia (A), oligodendrocytes (B) and neurons (C) in four independent glioma cohorts were distributed differently in glioma categories of different grades.

Figure S4

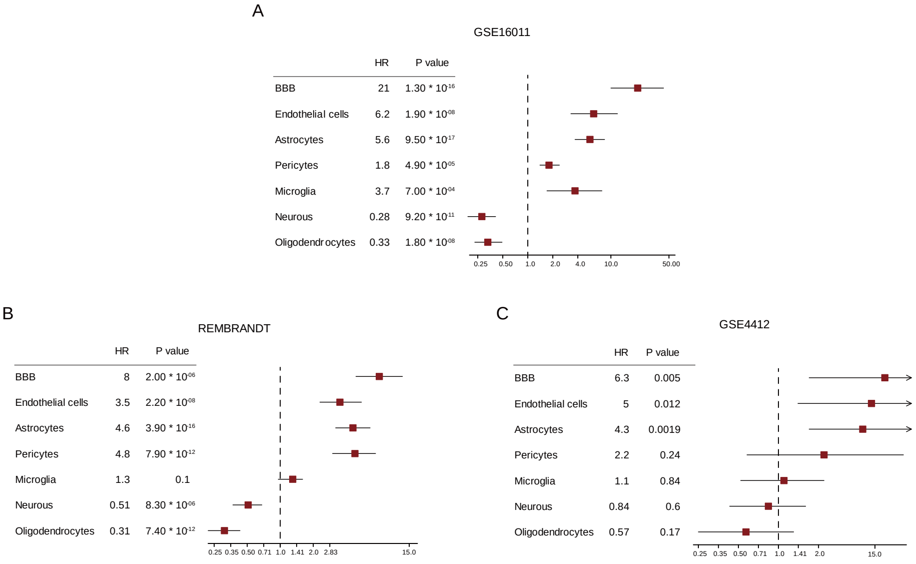

Figure S4: The impact of BBB score and six cell GSVA scores on patient survival in three independent glioma cohorts, GSE16011(A), REMBRANDT(B), GSE4412(C). The p-value is obtained from the test of the proportional risk (PH) assumption, and HR represents the risk ratio.

Figure S5

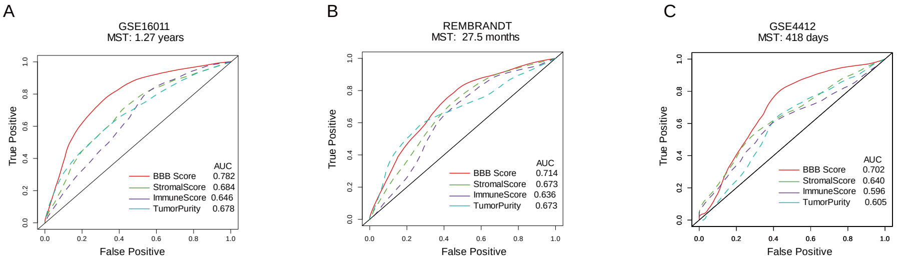

Figure S5: Comparison of survival prediction efficacy of BBB score and other microenvironment factors in three independent glioma cohorts, GSE16011(A), REMBRANDT(B), GSE4412(C).

Figure S6

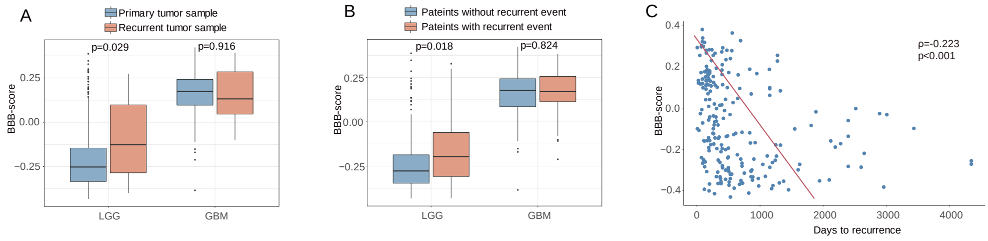

Figure S6: In the TCGA cohort, the differential distribution of BBB scores between primary and recurrent glioma samples(A), and recurrent and non recurrent patients after surgical resection(B). The correlation between the time from surgery to recurrence and BBB score in patients with recurrent glioma after surgical resection in TCGA cohort(C). The p values in Figures A and B are obtained by independent sample T test, and the p values in Figure C are obtained by Spearman correlation test.

Figure S7

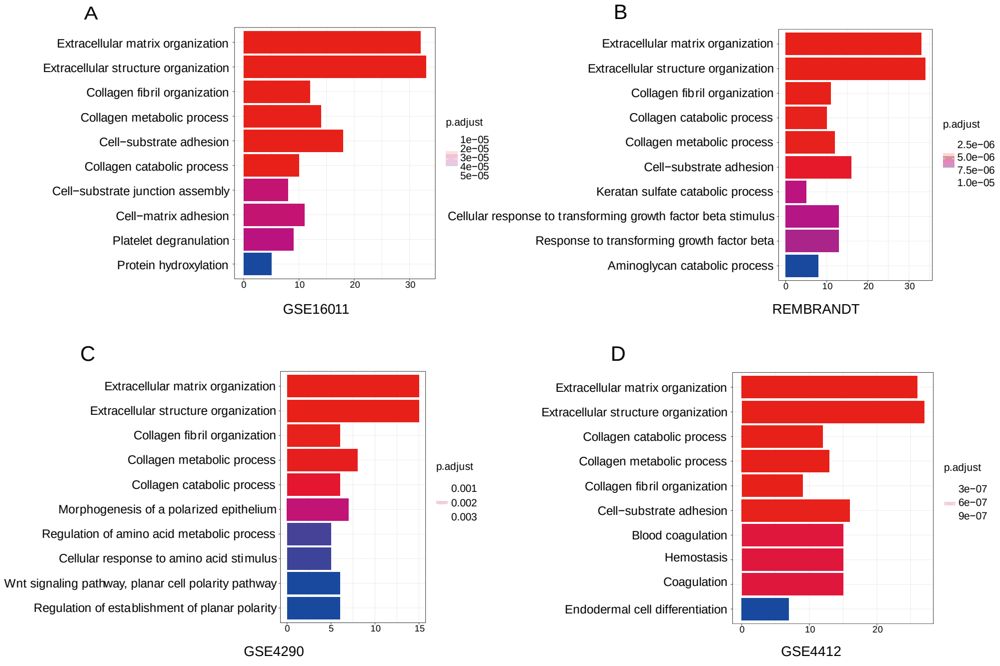

Figure S7: Biological processes and signal pathways related to BBB scores in GSE16011(A), REMBRANDT(B), GSE4290(C) and GSE4412(D) cohorts, the top 100 genes with the highest correlation with BBB score were the most significant top 10 pathways obtained by GO enrichment analysis(FDR corrected p-value).
